# Effects on the Thermo-Mechanical and Crystallinity Properties of Nylon 6,6 Electrospun Fibres Reinforced with One Dimensional (1D) and Two Dimensional (2D) Carbon

**DOI:** 10.3390/ma6083494

**Published:** 2013-08-14

**Authors:** Fabiola Navarro-Pardo, Gonzalo Martínez-Barrera, Ana Laura Martínez-Hernández, Víctor M. Castaño, José Luis Rivera-Armenta, Francisco Medellín-Rodríguez, Carlos Velasco-Santos

**Affiliations:** 1 Materials Science Postgraduate Studies, Faculty of Chemistry, Autonomous University of Mexico State, Km. 12 de la carretera Toluca-Atlacomulco, C.P. 50200, San Cayetano, Toluca, Estado de México, Mexico; E-Mails: faby.navpard@gmail.com (F.N.-P.); gonzomartinez02@yahoo.com.mx (G.M.-B.); 2 Laboratory of Research and Advanced Materials Development, Faculty of Chemistry, Autonomous University of Mexico State, Km. 12 de la carretera Toluca-Atlacomulco, C.P. 50200, San Cayetano, Toluca, Estado de Mexico, Mexico; 3 Centre of Applied Physics and Advanced Technology, National Autonomous University of Mexico, Boulevard Juriquilla No. 3001, C.P. 76230, Juriquilla, Santiago de Querétaro, Mexico; E-Mails: almh72@gmail.com (A.L.M.-H.); meneses@unam.mx (V.M.C.); 4 Division of Postgraduate Studies and Research, Technological Institute of Queretaro, Av. Tecnológico s/n, esq. Gral. Mariano Escobedo, Col. Centro Histórico, C.P. 76000, Santiago de Querétaro, Querétaro, Mexico; 5 Division of Postgraduate Studies and Research, Technological Institute of Madero City, Juventino Rosas y Jesús Ureta, Col. Los Mangos, C.P. 89440, Ciudad Madero, Tamaulipas, Mexico; E-Mail: jlriveraarmenta@itcm.edu.mx; 6 Faculty of Chemical Science, Autonomous University of San Luis Potosí, Av. Manuel Nava 6, Zona Universitaria, C.P. 78210, San Luis Potosí, San Luis Potosí, Mexico; E-Mail: francmr@uaslp.mx

**Keywords:** carbon nanotubes, graphene, polymer nanocomposites, crystallisation, mechanical properties

## Abstract

Electrospun one dimensional (1D) and two dimensional (2D) carbon based polymer nanocomposites are studied in order to determine the effect provided by the two differently structured nanofillers on crystallinity and thermo-mechanical properties of the nanofibres. The nanomaterials studied are pristine carbon nanotubes, oxidised carbon nanotubes, reduced graphene oxide and graphene oxide. Functional groups associated with the order structure of the polymers are analysed by infrared and Raman spectroscopies; the morphology is studied by scanning electron microscopy and the crystallinity properties are investigated by differential scanning calorimetry and X-ray diffraction. Differences in crystallisation behaviour between 1D and 2D carbon based nanofibres are shown by their crystallinity degree and their crystal sizes. The nanocomposite crystal sizes perpendicular to the plane (100) decrease with nanofiller content in all cases. The crystallinity trend and crystal sizes are in accordance with storage modulus response. The results also suggest that functionalisation favours interfacial bonding and dispersion of the nanomaterials within the polymer matrix. As a consequence the number of nucleating sites increases which in turn decreases the crystal size in the nanocomposites. These features explain the improved thermo-mechanical properties in the nanocomposites.

## 1. Introduction

Carbon nanotubes (CNTs) and graphene have attracted growing interest from a large variety of scientific communities investigating the properties and applications of these nanomaterials [1,2,3,4,5,6,7,8,9,10]. The great versatility of carbon nanomaterials arises from their physical, mechanical, electrical and thermal properties [1,2,3]. Their unique properties make them promising candidates for their use as advanced reinforcing fillers for high-strength, light-weight and functional polymer nanocomposites [11,12,13,14,15,16,17,18,19,20].

Polymer nanocomposites require homogeneous dispersion and strong interfacial interaction between the filler and the polymer matrix for the enhancement of mechanical and thermal properties [11]. Functionalisation in these materials provides efficient stress transfer between the polymer matrix and the nanometric carbon by preventing aggregation and providing a better dispersion of the nanomaterials in the polymer matrix [6,9]. Additionally, the functional groups at the surface of nanometric carbon create the strongest type of interfacial bonding with the polymer matrix [7,11].

The crystalline structure and the degree of crystallinity also play a crucial role in the properties of semi-crystalline polymers [13,14,17,18,19,20,21,22,23]. Nylon 6,6 is a semicrystalline polymer which has good thermal stability and mechanical strength and it is an important engineering thermoplastic [22,23,24]. This polymer is suitable for electrospinning processing due to its poly-electrolytic behaviour in acid solution [25]. The nanofibres obtained from electrospinning offer the possibility to incorporate active components on a nanoscale [16,26]. Furthermore, it is well known that the addition of carbon nanomaterials can promote the crystallisation process of polymers due to their nucleating effects [13,14,16,17,18,19].

The enhancement of the nanocomposite mechanical properties due to functionalisation of 1D and 2D carbon has been widely studied [11,12,13,14]. The impact on crystallisation that both carbon nanomaterials provide in polymer nanocomposites has also been investigated [13,14,17,18,19,20,21]. Rong* et al.* found that functionalised CNTs have a better performance in improving the mechanical properties of poly (ether ether ketone) when compared to pristine CNTs [13]. However at high content of functionalised CNTs the crystallisation rate decreases [13,14]. Yun* et al.* have shown that the re-crystallisation temperatures and Young’s modulus of polypropylene were increased gradually with increasing alkylated graphene oxide content [18]. Xu* et al.* worked on the crystallisation of poly (L-lactide) induced by CNTs and graphene [19]. They found that when increasing the CNT content the nanocomposite crystallisation rate was enhanced but the reverse situation was found for graphene nanocomposites. Our research group worked with amine modified CNTs and graphene in electrospun fibres [20]. Amino functionalised CNTs resulted in better mechanical properties at the lowest loading; on the other hand amino functionalised graphene showed improved reinforcing effect by increasing the nanofiller loading. This trend was consistent with the crystallinity of the nanofibres.

To-date, several studies describe the fabrication of nanocomposite fibres with improved thermo-mechanical properties derived from the incorporation of nanometric carbon. To our knowledge there is no previous research that focuses on the crystallinity and mechanical properties of pristine and functionalised 1D and 2D structured carbon nanocomposites obtained at the same conditions and in the same matrix. The goal of this study is to understand the influence of these novel carbon nanomaterials with different structure (1D and 2D) and chemistry, as reinforcing and nucleating fillers in a polymer nanocomposite processed at the same conditions.

## 2. Results and Discussion

**Figure 1:**
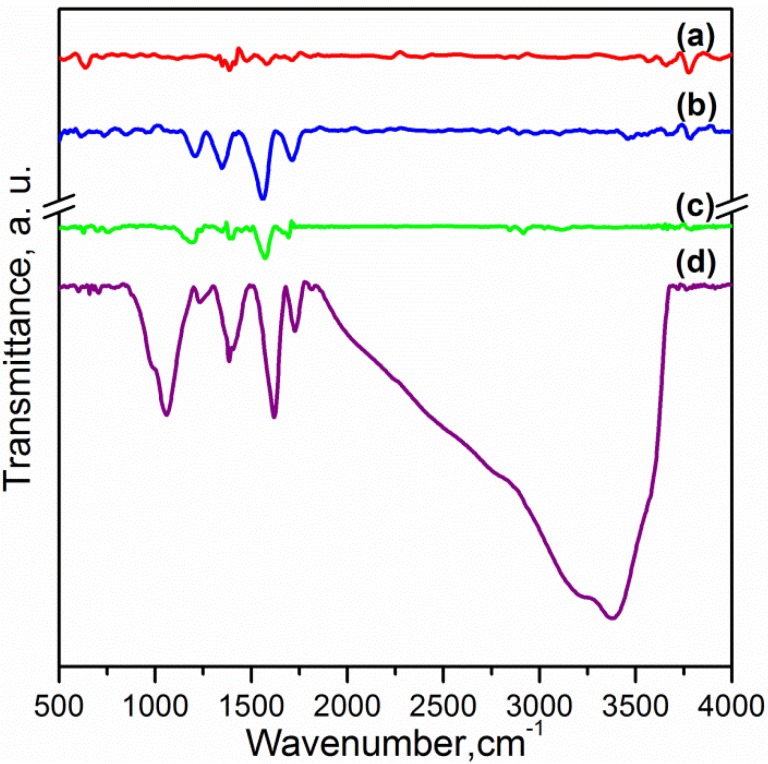
Fourier transform infrared spectroscopy (FTIR) spectra of: (**a**) Carbon nanotubes (CNTs); (**b**) oxidised carbon nanotubes (OCNTs); (**c**) reduced graphene oxide (RGO) and (**d**) graphene oxide (GO).

Fourier transform infrared (FTIR) spectra of pristine CNTs and oxidised carbon nanotubes (OCNTs) are shown in Figure 1. The OCNTs present peaks at 1208 cm^−1^, 1350 cm^−1^ and 1713 cm^−1^ corresponding to the characteristic C–O, O–H and C=O stretching vibrations of carboxylic groups [13,15]. The peak at 1560 cm^−1^ is attributable to the influence of the C=O vibration of carboxilate groups produced in the oxidation process [14].

Figure 1 also shows the typical peaks of graphene oxide (GO) for C–O stretching of epoxy and alcoxi at 1058 cm^−1^ and 1240 cm^−1^ respectively [6,7]. The peaks at 1395 cm^−1^ and 1728 cm^−1^ reflect the carboxylic acid groups due to the O–H in-plane deformation for the former and C=O stretching vibration for the latter [5,7,8]. The peak at 1616 cm^−1^ is attributable to skeletal vibrations of unoxidised graphitic domains and also to the stretching vibration of intercalated water. This is also observed in the broad peak around 3300 cm^−1^ and is a feature of the O–H stretching vibration [5,6,8]. Several studies have reported that complete water removal from GO is practically impossible [8]. After reduction the peaks of oxygen functional groups vanished. The weak peaks at 1030 cm^−1^, 1187 cm^−1^ and 1333 cm^−1^ are features of the C–N stretching [9]. The peak at 1698 cm^−1^ is ascribed to the C=O stretching [9]. This indicates a few amine groups adhered to the graphene surface. The peak at 1570 cm^−1^ is in accordance with the C=C vibrations contributed from aromatic ring mode [10].

**Figure 2:**
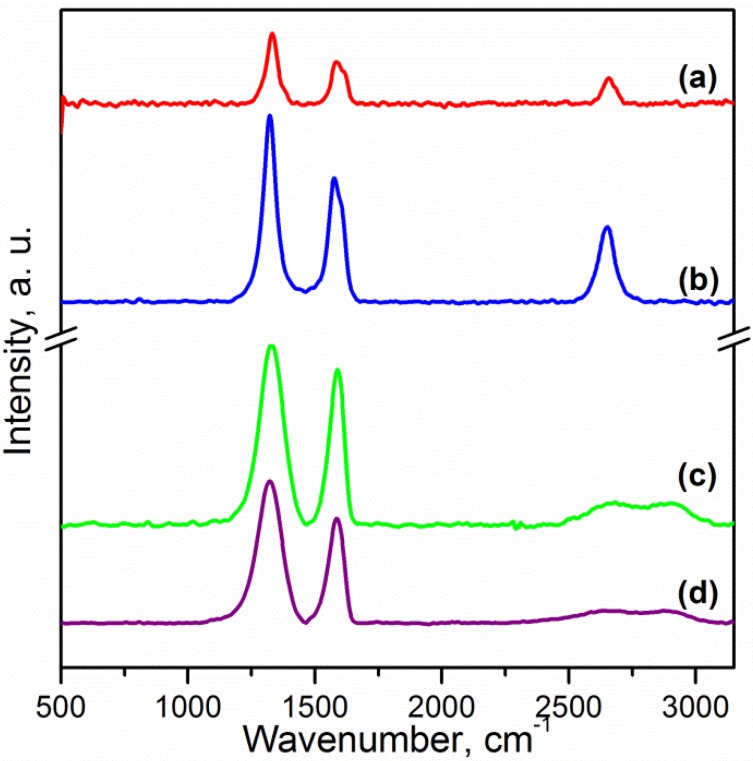
Raman spectra of: (**a**) CNTs; (**b**) OCNTs; (**c**) RGO and (**d**) GO.

Raman spectroscopy is a suitable technique to study the ordered/disordered crystal structures of carbonaceous materials. These materials show similar features in the 800–2000 cm^−1^ region, which is of interest for CNTs and graphene [2]. The usual characteristics of carbon materials in Raman spectra are the G and the D bands. The G band (~1580 cm^−1^) is usually attributed to the E_2g_ phonon of C sp^2^ atoms and the D band (~1320 cm^−1^) is due to the breathing mode of κ-point phonons of A_1g_ symmetry [3]. The D band reflects the local defects and disorders particularly located at the edges of graphene. The Raman spectra of carbon nanotubes and graphene are shown in Figure 2. All spectra display the G and D bands. The intensity ratio of the D and the G band (I_D_/I_G_) for CNTs and OCNTs were 1.53 and 1.40. This has been attributed to sp^2^ C atoms converted to sp^3^ C atoms at the surface of the CNTs after functionalisation [12]. The values of the I_D_/I_G_ ratio for reduced graphene oxide (RGO) and GO are 1.32 and 1.14 respectively. A larger I_D_/I_G_ peak intensity ratio has been assigned to a decrease in the average size of the sp^2^ domains upon reduction of GO, meaning that the newly created graphitic domains are smaller but more numerous in number [27]. Moreover a 5 cm^−1^ redshift in the G band of RGO was observed. This feature shows the successful reduction of graphene oxide [7]. Another fingerprint of carbon nanomaterials is the 2D band (sometimes labelled as D′ band) at about 2650 cm^−1^. The shape, position and intensity relative to the D band of this peak depend markedly on the number of layers in graphene [28]. This band is difficult to observe in the spectra; however this weak and broad peak is in accordance with studies of chemically reduced graphene [6].

**Figure 3:**
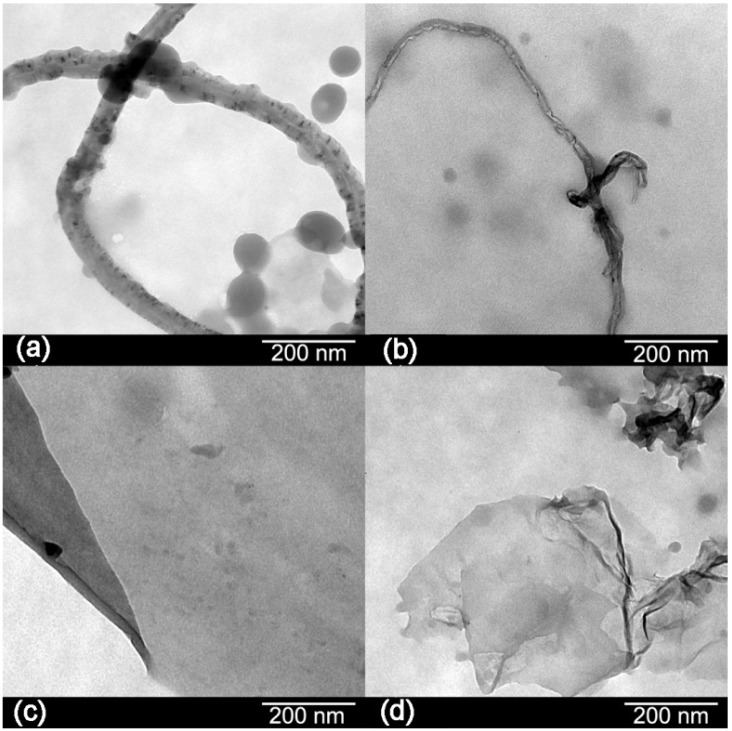
Transmission electron microscopy (TEM) images of: (**a**) CNTs; (**b**) OCNTs; (**c**) RGO and (**d**) GO.

The morphologies of the carbon materials are shown in Figure 3. Transmission electron microscopy (TEM) images showed black spots in the structure of the CNTs indicating impurities. After oxidation, there are few black spots in their structure which means that most of the impurities have been removed [8]. Graphene sheets appear as a transparent thin paper structure with some folds. Their resemblance of crumpled silk veil waves that were corrugated and scrolled is intrinsic to graphene sheets [6]. Wrinkling in GO is due to defects and attraction between oxygen groups formed on the surface of the sheet [10].

**Figure 4:**
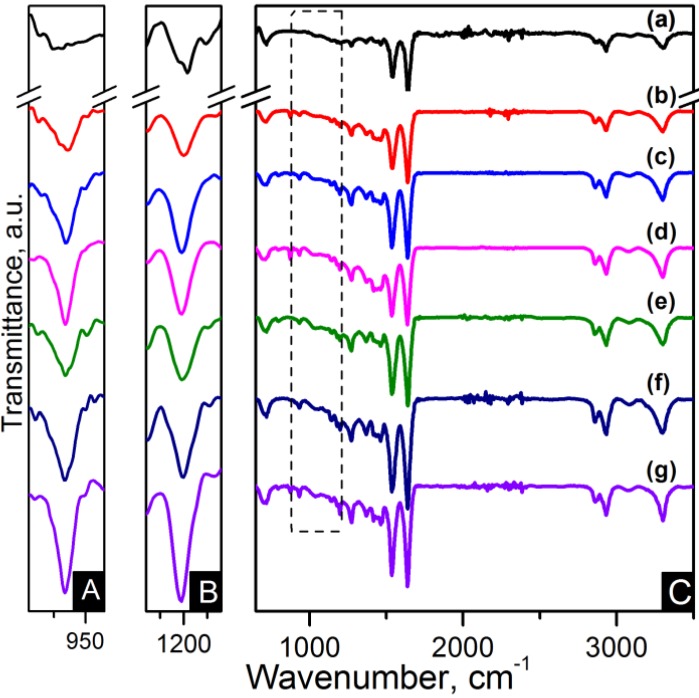
FTIR spectra of (**A**) zoom-in of ~935 cm^−1^ peak; (**B**) zoom-in of ~1200 cm^−1^ peak and (**C**) region of 900–3500 cm^−1^ of the samples: (**a**) pure PA66; (**b**) PA66/01CNTs; (**c**) PA66/05CNTs; (**d**) PA66/10CNTs; (**e**) PA66/01OCNTs; (**f**) PA66/05OCNTs and (**g**) PA66/10OCNTs.

**Figure 5:**
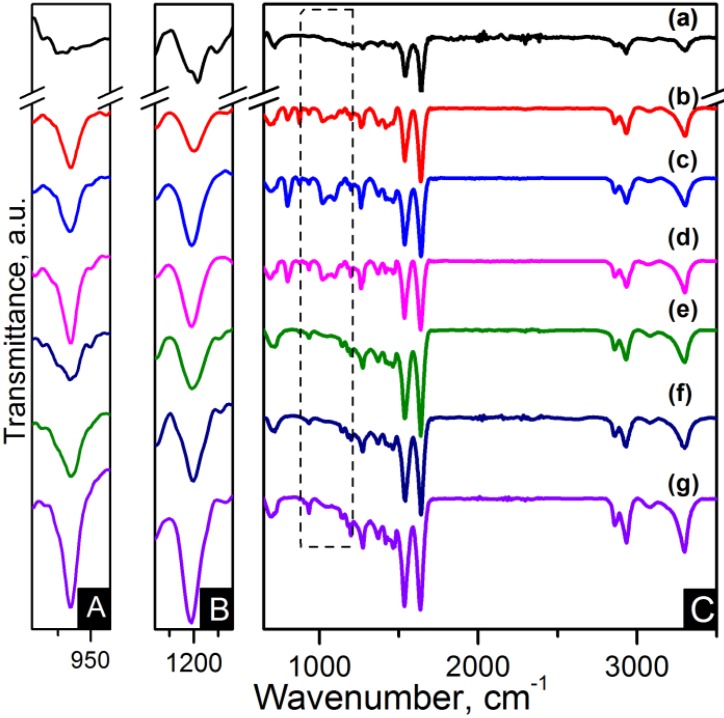
FTIR spectra of (**A**) zoom-in of ~935 cm^−1^ peak**;** (**B**) zoom-in of ~1200 cm^−1^ peak and (**C**) region of 900–3500 cm^−1^ of the samples: (**a**) pure PA66; (**b**) PA66/01RGO; (**c**) PA66/05RGO; (**d**) PA66/10RGO; (**e**) PA66/01GO; (**f**) PA66/05GO and (**g**) PA66/10GO.

FTIR spectroscopy has been applied for identification of the basic structural units present in nylon 6,6 electrospun fibres and carbon based nanocomposites. The nanocomposites were labelled as listed in Table 1. The assignments of all the fundamental bands are given in Table 2 [29,30,31]. The FTIR spectra of the electrospun nanofibres are presented in Figure 4 and Figure 5. The FTIR spectra show the characteristic bands of nylon 6,6: N–H deformation and C–N stretching of amide II band at ~1535 cm^−1^, C=O stretching, N–H stretching vibration of amide I band at ~1635 cm^−1^ and N–H stretching at ~3300 cm^−1 ^ [29,32]. Infrarred (IR) spectra are sensitive to the conformation and packing of chain molecules, and this sensitivity has been widely exploited to characterise semicrystalline polymers in terms of their crystallinity. Nylon 6,6 has two characteristic crystalline peaks at 935 cm^−1^ and 1200 cm^−1^ [29,32,33]. The addition of carbon nanomaterials changed the peak intensities compared to pure polymer indicating enhanced crystallinity as seen in the zoom-in of the crystalline peaks of Figure 4 and Figure 5.

**Table 1:** Nomenclature of the electrospun fibres.

Type of nanofillers	Nanofiller content		
	0.1 wt %	0.5 wt %	1.0 wt %
Carbon nanotubes (CNTs)	PA66/01CNTs	PA66/05CNTs	PA66/10CNTs
Oxidised carbon nanotubes (OCNTs)	PA66/01OCNTs	PA66/05OCNTs	PA66/10OCNTs
Reduced graphene oxide (RGO)	PA66/01RGO	PA66/05RGO	PA66/10RGO
Graphene oxide (GO)	PA66/01GO	PA66/05GO	PA66/10GO

**Table 2:** Fourier transform infrared spectroscopy (FTIR) band assignments in Nylon 6,6 [26,30,33].

Band [cm^−1^]	Assignments
~934	Crystalline peak, amide axial deformation (C–C=O)
1033–1043 and 1063–1066	Triclinic structure, skeleton axial elongation (C–C)
1140–1146	Angular deformation out of plane of carbonyl groups
~1202	Crystalline peak: symmetrical angular deformation out of plane, amide III.
~1220	Angular deformation out of plane,(H–N–C=O)
1300–1305	Angular deformation out of plane, N–H
~1370	C–N axial deformation
~1440	CH_2_ deformation
1535–1555	C–N axial deformation and CO–N–H angular deformation, amide II
~1640	C=O axial deformation, amide I
~2858	CH_2_ axial deformation
~2950	CH_2_ axial deformation
~3080	N–H angular deformation in the plane
~3300	Free N–H axial deformation

**Figure 6:**
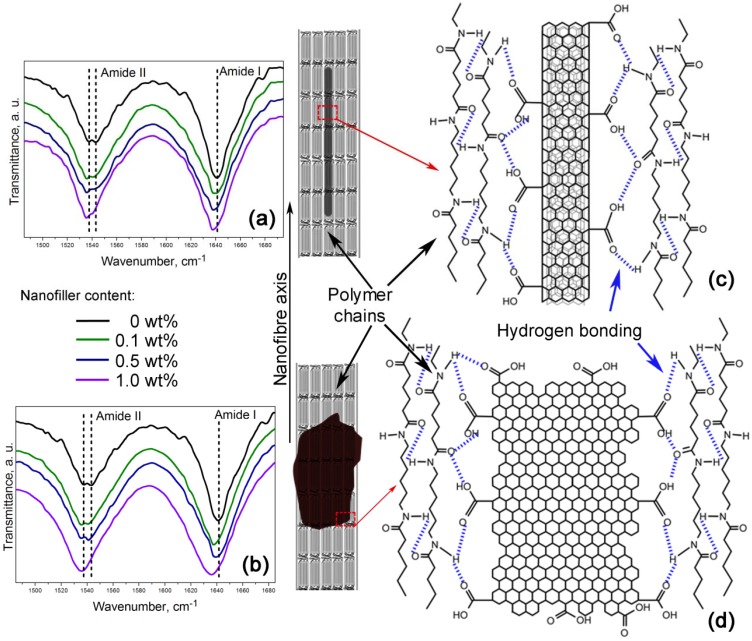
Nanofibres intramolecular bonding in the (**a**,**c**) PA66/OCNTs and the (**b**,**d**) PA66/GO nanocomposites.

Furthermore, Figure 6a,b shows a zoom-in of the 1485–1695 cm^−1^ FTIR region indicating the amide I and the amide II bands. These bands are of particular interest because they are related to hydrogen bonding [34,35]. Lu *et al.* studied the amide I band in nylon 6,6/clay nanocomposites, which is composed of two components, attributable to the ordered and disordered hydrogen-bonded carbonyl groups [34]. They found the former had a higher strength and that their frequency of stretching was lower than that of the latter.

Our nanocomposites showed an increase of intensity in the amide I and the amide II bands as the nanofiller loading increased, which is also indicative of the formation of hydrogen bonding [35]. The spectra also show that amide II band splits in two components, as evidenced by the peaks at ~1537 cm^−1^ and ~1544 cm^−1^. At lower frequency the peak becomes more intense as the content of OCNTs and GO is increased. The amide II band in PA66/10GO nanofibres lacks of this splitting because the component of the ordered hydrogen-bonded groups overlap the component of the ones in the disordered state. These features demonstrate the hydrogen bonding between the nanofillers and the polymer, as displayed in the schemes of Figure 6c,d.

**Figure 7:**
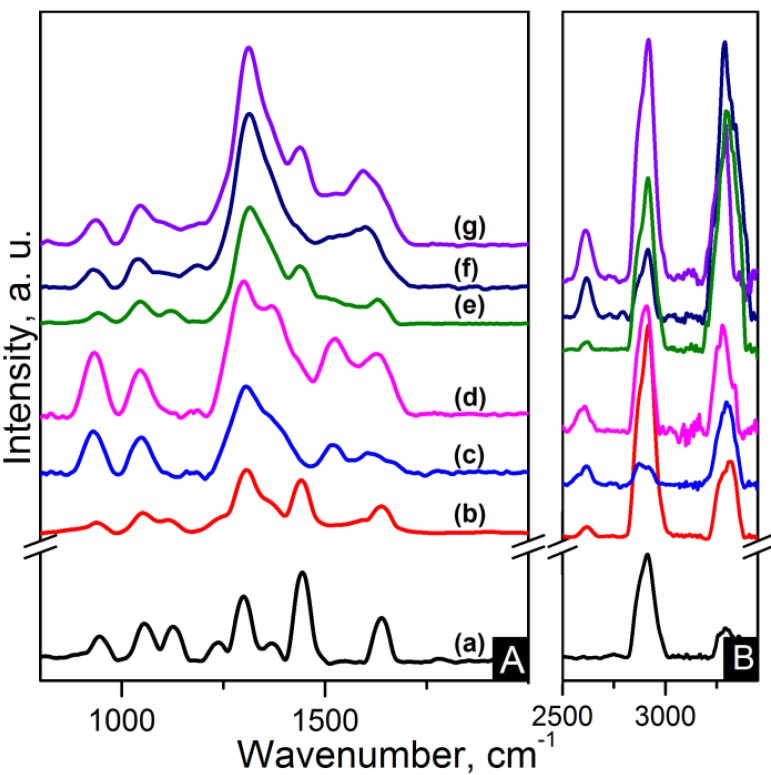
Raman spectra of the (**A**) 800–2000 cm^−1^ region and (**B**) zoom-out of the 2800–3350 cm^−1^ region of the samples: (**a**) pure PA66; (**b**) PA66/01CNTs; (**c**) PA66/05CNTs; (**d**) PA66/10CNTs; (**e**) PA66/01OCNTs; (**f**) PA66/05OCNTs and (**g**) PA66/10OCNTs.

**Figure 8:**
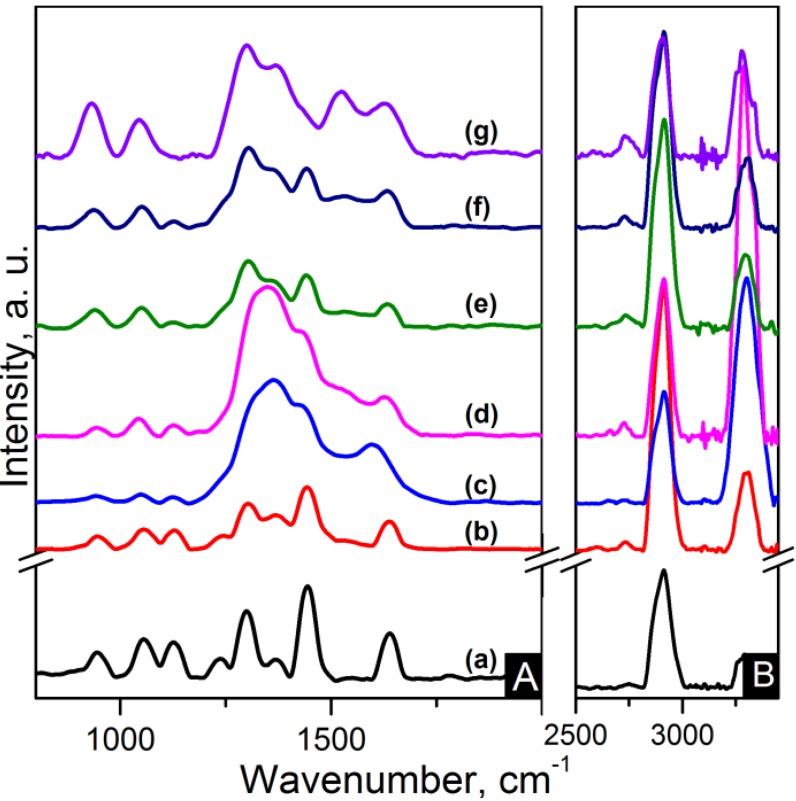
Raman spectra of the (**A**) 800–2000 cm^−1^ region and (**B**) zoom-out of the 2800–3350 cm^−1^ region of the samples: (**a**) pure PA66; (**b**) PA66/01RGO; (**c**) PA66/05RGO; (**d**) PA66/10RGO; (**e**) PA66/01GO; (**f**) PA66/05GO and (**g**) PA66/10GO.

IR and Raman spectroscopy are complementary to each other. Strong IR bands are related to polar functional groups whereas non-polar functional groups give rise to strong Raman bands [33]. Raman spectra of the nanocomposites are shown in Figure 7 and Figure 8. C–C=O stretching is found at ~945 cm^−1^. The region between 1000 cm^−1^ and 1170 cm^−1^ is characteristic of the stretching of the C–C skeletal backbone structure. The peak at ~1235 cm^−1^ is due to N–H wagging. Peaks ranging from 1265 to 1500 cm^−1^ are attributable to bending vibrations of the CH_2_ groups. Carbon based nanocomposites spectra show overlapping of the D band in this region. G band also is also overlapped in the zone between 1480 cm^−1^ and 1700 cm^−1^ where amide I band (C=O) is located at ~1640 cm^−1^. The 2D band appears at ~2620 cm^−1^ for CNT based nanocomposites and features as a weak wide band in the region of 2500–2800 cm^−1^ for the graphene based nanocomposites. The CH_2_ asymmetric and symmetric stretching and the N–H stretching appear as strong broad bands in the 2800–3000 cm^−1^ region for the former and in the 3200–3400 cm^−1^ region for the latter [33].

**Figure 9:**
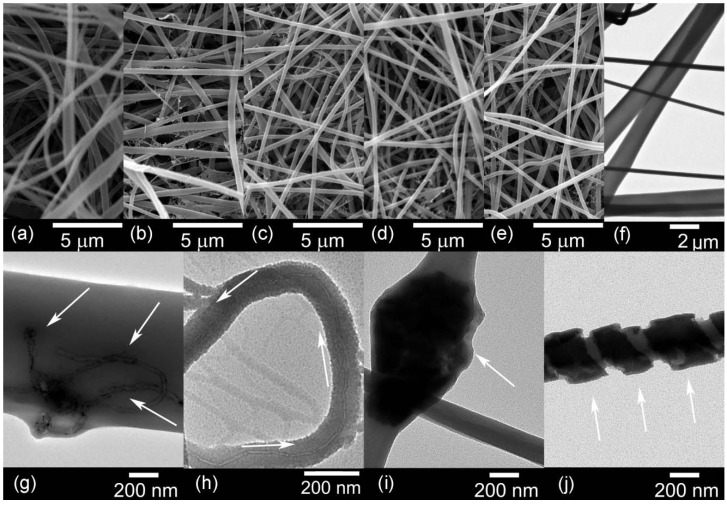
Scanning electron microscopy (SEM) and TEM images of: (**a**,**f**) pure PA66; (**b**,**g**) PA66/10CNTs; (**c**,**h**) PA66/10OCNTs; (**d**,**i**) PA66/10RGO and (**e**,**j**) PA66/10GO.

The diameters of the fibres were obtained from the scanning electron microscopy (SEM) images, as displayed in Table 3. Nylon 6,6 nanofibres had an average diameter of 633 nm. The addition of nanometric carbon to the polymeric matrix resulted in a decrease of the nanofibre diameters. Physical properties such as viscosity and conductivity influence the morphology of the nanofibres [31]. The incorporation of carbon nanomaterials increased the viscosity and improved the conductivity of the solution. While a more viscous solution produces thicker fibres, the increase in conductivity favours the stretching of thinner fibres [26]. These parameters are the reason of the variable diameters in the samples. Figure 9a–e shows the morphology of the nanofibres containing the highest nanofiller loading.

**Table 3:** Average diameters of the electrospun fibres.

Nanofiller wt %	PA66/CNTs [nm]	PA66/OCNTs [nm]	PA66/RGO [nm]	PA66/GO [nm]
0.1	243	427	315	306
0.5	332	357	260	271
1.0	410	325	428	302

TEM images are included in Figure 9g–j, the micrographs of the nanocomposites show the dispersion of the nanofillers in the matrix. Figure 9g shows that at the highest loading pristine CNTs are agglomerated in the nanofibre, as indicated by the arrows. On the other hand, Figure 9h shows the OCNTs are aligned along the fibre. Figure 9i,j shows the graphene nanomaterials are embedded within the polymer and GO followed a pattern along the fibre.

**Figure 10:**
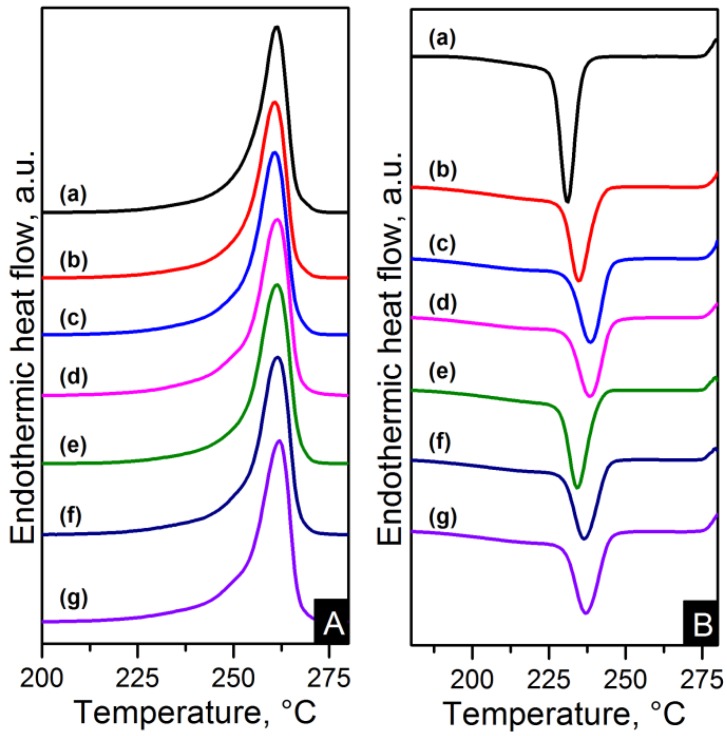
Differential scanning calorimetry (DSC) (**A**) heating and (**B**) cooling thermograms of: (**a**) pure PA66; (**b**) PA66/01CNTs; (**c**) PA66/05CNTs; (**d**) PA66/10CNTs; (**e**) PA66/01OCNTs; (**f**) PA66/05OCNTs and (**g**) PA66/10OCNTs.

**Figure 11:**
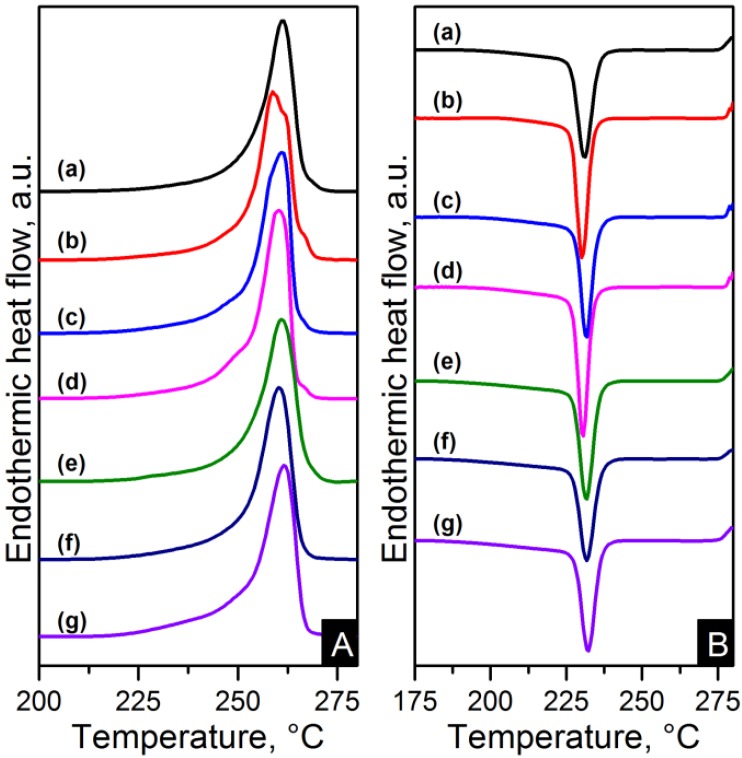
DSC (**A**) heating and (**B**) cooling thermograms of: (**a**) pure PA66; (**b**) PA66/01RGO; (**c**) PA66/05RGO; (**d**) PA66/10RGO; (**e**) PA66/01GO; (**f**) PA66/05GO and (**g**) PA66/10GO.

The melting and crystallisation curves obtained from differential scanning calorimetry (DSC) are shown in Figure 10 and Figure 11. There is a slight difference in the melting peaks as seen in Figure 10A and Figure 11A. Both electrospun nanocomposites and nylon 6,6 showed a broad melting peak at ~260 °C, indicating the melting of α-crystals [22]. There is also a slight broad shoulder on the low temperature side at ~250 °C which is more noticeable in the RGO nanocomposites. This has been attributed to morphological changes in the crystallite or the melting of small and less stable crystalline units [23,24,29]. The crystal characteristics found in the nanocomposites due to the different nanometric structure of CNTs and graphene will be discussed in more detail later. The degree of crystallinity (X*_c_*) was calculated as shown in Equation (1).

(1) $$X_{c}=\frac{\Delta H_{m}}{\Delta {H^{0}}_{m}}\times 100\%$$

where *ΔH_m_* and *ΔH_m_*^0^ (197 J/g) are the enthalpies of the nanocomposite and purely crystalline nylon 6,6 respectively [23]. The crystallinity properties are summarised in Table 4. The electrospun nanocomposites resulted in higher crystallinity than pure polymer. These results are consistent with earlier studies of carbon based nanocomposites [16,21]. The crystallinity changes from ~39% to ~44% for the PA66/10GO sample compared to nylon 6,6. The higher results in degree of crystallinity when increasing the content indicate the induced crystallisation due to the CNTs and graphene materials.

**Table 4:** Crystallisation properties of the electrospun fibres.

Sample	X_c _^a^ [%]	CI ^b^	L_(100)_^ c^ [nm]	L_(010/110)_^ d^ [nm]
Pure PA66	39.4	0.38	6.45	3.41
PA66/01CNTs	39.0	0.39	6.32	3.18
PA66/05CNTs	41.4	0.40	6.06	3.35
PA66/10CNTs	41.8	0.42	6.00	3.14
PA66/01OCNTs	40.8	0.39	6.12	3.09
PA66/05OCNTs	41.3	0.40	5.63	3.15
PA66/10OCNTs	42.1	0.44	5.61	3.11
PA66/01RGO	41.5	0.42	5.44	2.89
PA66/05RGO	42.5	0.43	5.43	2.98
PA66/10RGO	43.5	0.44	5.18	2.92
PA66/01GO	41.8	0.40	5.60	3.17
PA66/05GO	42.6	0.42	5.10	3.08
PA66/10GO	43.8	0.44	5.01	2.93

^a^ Crystallinity; ^b^ crystallinity index; ^c^ crystal size perpendicular to the (100) plane; ^d^ crystal size perpendicular to the (010/110) plane.

The crystallisation thermograms of the nanocomposites showed higher crystallisation temperatures (T*_c_*) for the CNT based nanocomposites than nylon 6,6. This is explained by the increased amount of nuclei crystallising in the matrix due to addition of CNTs. The one-dimensional carbon material also blocked the nylon 6,6 chains mobility which resulted in an accelerated nucleation process [13].

CNT thermograms displayed an average increase in T*_c_* of 6 °C compared to pure polymer. The average T*_c_* in OCNT nanocomposites resulted in an increase of 4.6 °C. This behaviour indicates that functionalisation of carbon nanomaterials slightly weakens the heterogeneous nucleation effect of the nanotubes as is in agreement with earlier studies [13,14]. On the other hand, graphene based nanocomposites showed comparable or slightly higher T*_c_* to that of nylon 6,6. A similar nucleating effect of the graphene compared to CNTs has been found in polypropylene and poly(L-lactide) nanocomposites [18,19]. This behaviour has been explained by the considerable large surface area of the graphene sheets where the polymer chains need more time to adjust their conformations making the induction of crystallisation slower [19].

**Figure 12:**
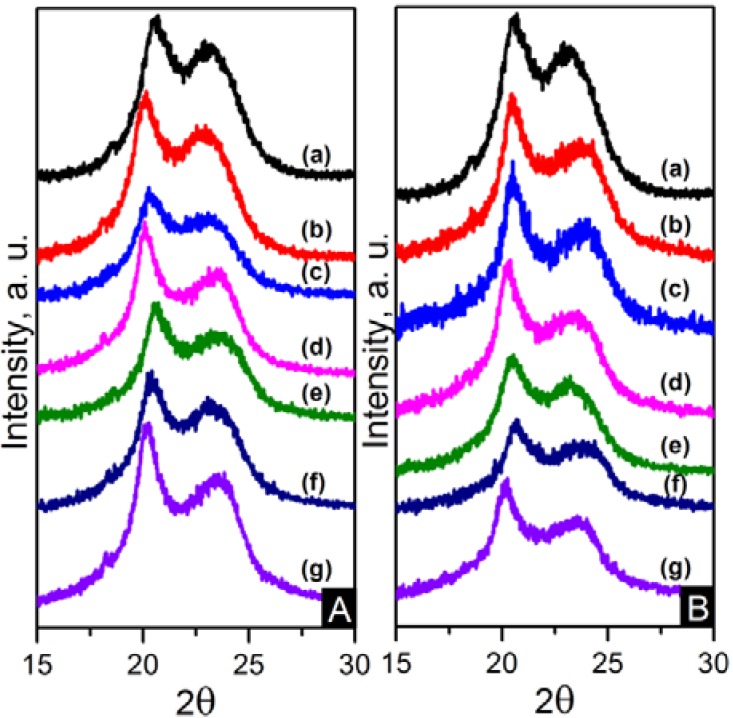
(**A**) WAXD patterns of: (**a**) pure PA66; (**b**) PA66/01CNTs; (**c**) PA66/05CNTs; (**d**) PA66/10CNTs; (**e**) PA66/01OCNTs; (**f**) PA66/05OCNTs and (**g**) PA66/10OCNTs. (**B**) WAXD patterns of: (**a**) pure PA66; (**b**) PA66/01RGO; (**c**) PA66/05RGO; (**d**) PA66/10RGO; (**e**) PA66/01GO; (**f**) PA66/05GO and (**g**) PA66/10GO.

The crystalline structure of the nanocomposites was also characterised using wide angle X-ray diffraction (WAXD). WAXD patterns are displayed in Figure 12. Two peaks observed at approximately 20.5° and 23.5° are consistent with the diffraction of (100) and (010/110) crystalline planes of α-crystals [22]. The diffraction pattern was decomposed using peak-fitting of Gaussian functions in Origin^®^ 8.5 in order to obtain a broad amorphous halo and sharp peaks from reflections of the crystalline peaks and to evaluate the crystallinity index (CI) as shown in Equation (2).

(2) $$CI=\frac{A_{c}}{A_{c}+A_{a}}$$

where *A_c_* is the integrated area underneath the crystalline peaks and *A_a_* is the integrated area of the amorphous halo. The values of CI are also shown in Table 4. These values are in accordance with the DSC crystallinity results.

The unit cell of α-crystals in nylon 6,6 is triclinic [22,23,24]. The two strong diffraction signals are a project value of interchain distance within the hydrogen-bonded sheet (100) and the (010/110) signal represents the intersheet distance [24]. The crystallite size perpendicular to the diffraction (hkl) plane, L_hkl_ in nanometres, can be obtained by applying Scherrer’s equation, displayed in Equation 3.

(3) $$L_{hkl}=\frac{k\lambda }{\beta \cos\theta }$$

where k is the Scherrer factor (0.9 for Gaussian function), λ is the X-ray wavelength, $\beta ={\left(B^{2}-b_{0}^{2}\right)}^{1/2}$ is the pure line breadth, B is a measured half width of the experimental peak, $b_{0}$ is the instrumental broadening factor which is 0.17 for the diffractometer employed , and θ is the Bragg angle. The crystallite sizes L_(100)_ and L_(010/110)_ of nanocomposites are smaller compared to nylon 6,6. Table 4 reveals the crystallite size decreases with content due to the suppressed crystal growth caused by the interaction between nylon 6,6 and nanometric carbon. This means that the degree of crystal perfection decreased as the content increased [24,34]. Crystal size values of graphene based nanofibres are lower than crystal size of CNT based nanocomposites. Functionalisation also has an impact on this property; the effect is more noticeable in the OCNT nanofibres. RGO and GO nanocomposites showed similar results. The nanofibre crystal sizes are as expected based on studies of nylon 6,6 fibres and nylon 6,6 nanocomposites [24,34]. Crystallisation in nylon 6,6/graphene nanocomposites has been explained as a two factor controlled process. One factor is the nucleating effect of graphene and the other factor is the retarded migration and diffusion of polymer molecular chains to the surface of the nucleus which constrain crystal growth [17,19]. It has also been proposed that the growing crystals on graphene surface could show multiple orientations, which might contact the adjacent single crystals and suppress this process [19]. These explanations serve as evidence of the different crystal size between 1D and 2D carbon based nanocomposites studied in this paper. RGO nanocomposites showed the most reduced crystal size values, confirming the presence of the smaller crystallites melting at lower temperatures in the heating thermograms. In spite of these results, the values of GO nanocomposites show a trend of decreasing crystal size with content in both directions. This is another indication of the superior dispersion of GO in the nanofibres. A decrease in crystallite size is likely to favour mechanical properties [34].

**Figure 13:**
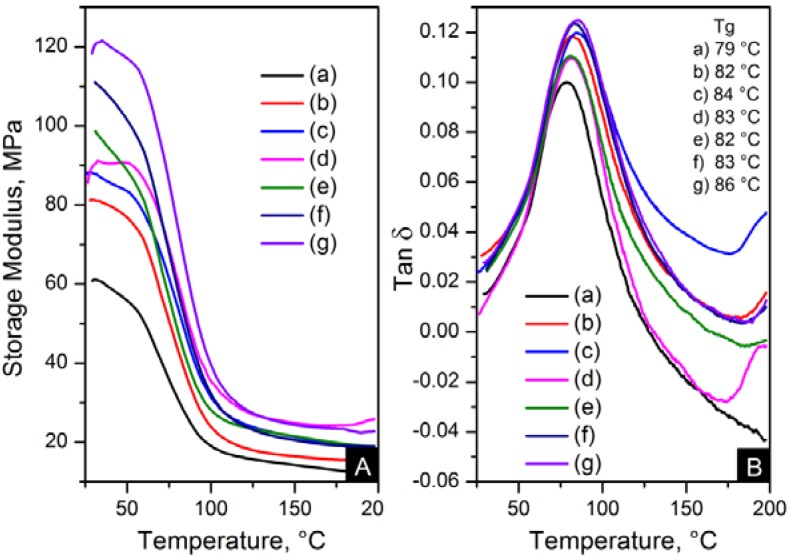
Dynamic mechanical analyser (DMA) results; (**A**) storage modulus and (**B**) Tanδ of: (**a**) pure PA66; (**b**) PA66/01CNTs; (**c**) PA66/05CNTs; (**d**) PA66/10CNTs; (**e**) PA66/01OCNTs; (**f**) PA66/05OCNTs and (**g**) PA66/10OCNTs.

**Figure 14:**
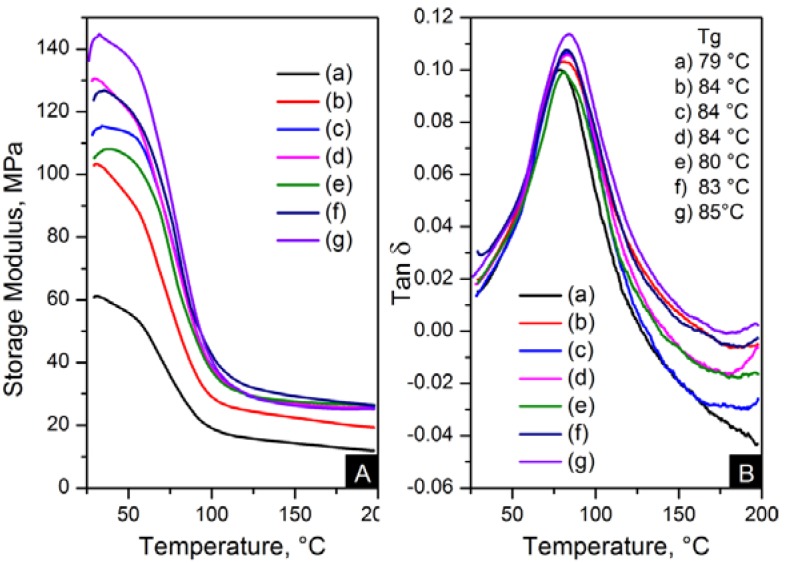
DMA results; (**A**) storage modulus and (**B**) Tanδ of: (**a**) pure PA66, (**b**) PA66/01RGO; (**c**) PA66/05RGO; (**d**) PA66/10RGO; (**e**) PA66/01GO; (**f**) PA66/05GO and (**g**) PA66/10GO.

The reinforcement effect of the nanofillers in nylon 6,6 is seen in dynamic mechanical analyser (DMA) results, displayed in Figure 13 and Figure 14. The addition of nanomaterials affected the stiffness of the polymer. As expected, the storage modulus was enhanced compared to pure polymer. This trend is consistent with an increase in content and the decrease of crystal size, as seen in Table 4.

The storage modulus of the OCNT nanocomposites was almost the double than that of pristine CNT nanocomposites. The nanofibres containing 1 wt % OCNTs resulted in a 97% increase in the mechanical response. A more significant improvement in the mechanical properties resulted from the graphene based nanocomposites. Higher modulus values were obtained for GO nanocomposites, where storage modulus was improved by 139% compared to nylon 6,6. The enhanced mechanical properties can be explained on the basis of crystal features imposed by the structure of nanometric carbon and the reinforcing effect of nanomaterials. This is attributed due to the superior dispersion of the nanomaterials in the matrix provided by their functionalisation. It was evident that the improved dispersion provided by functionalisation resulted in a more homogeneous confinement of the crystals of nylon 6,6. This was demonstrated by the reduction in crystal size after incorporation of OCNTs and GO. The graphene based nanocomposites show the smallest crystal size values than the rest of the nanocomposites; this explains the enhanced mechanical response. Furthermore, it is supposed that the large surface area of graphene provided by its 2D structure play an important role to generate better interaction with the polymer favouring the stress transfer from the nanofiller to the matrix. Therefore, the graphene nanocomposites resulted in superior mechanical properties.

Figure 13B and Figure 14B show the glass transition temperatures (T*_g_*) obtained from the tanδ maximum. The values of T*_g_* were higher in the nanocomposites than nylon 6,6 nanofibres. This property was increased up to 7 °C for PA66/10OCNTs. T*_g_* in GO nanocomposites showed a 6 °C increase for the maximum filler content. This increase in T*_g_* results from the presence of nanomaterials which restricted the molecular mobility on the matrix. The free volume of the nylon 6,6 chains is influenced by the interaction between nanofiller and the matrix [16]. The improvement in T*_g_* is a feature of the confinement imposed by the nanomaterials in nylon 6,6 molecular segments.

## 3. Experimental Section

### 3.1. Nanomaterials Functionalisation

Chemical vapour deposition multiwalled carbon nanotubes (Sunnano Company, China), with 10–30 nm in outer diameter, 1–10 μm length and purity above 80%, were refluxed for three hours at 80 °C in a 3:1 molar solution of nitric acid (HNO_3_, 70%, Sigma-Aldrich) and sulphuric acid (H_2_SO_4_, 98%, J.T. Baker). The solution was filtered and washed with distilled water until neutral pH. Finally, the oxidised carbon nanotubes were dried overnight at 80 °C.

GO was prepared by oxidation of graphite using the modified Hummers method [4]. 23 mL of H_2_SO_4_ were added into a reaction flask submerged in an ice bath and kept there until it reached 0 °C. 1 g graphite (No. 70230, Electron Microscopy Science) and 3 g potassium permanganate (KMnO_4, _Merck) were added slowly followed by stirring at 35 °C for two hours and then diluted with 46 mL of distilled water for 15 min under stirring. After that a solution of 5 mL of hydrogen peroxide (H_2_O_2_, 30%, J.T. Baker) in 135 mL of distilled water was added to reduce residual KMnO_4_. A solution of 2.5 mL hydrochloric acid (HCl, 37%, Sigma-Aldrich) in 100 mL of distilled water was added to remove metal ions followed by filtration with excess water to remove the acid. Finally the graphite oxide was dried overnight at 60 °C. The resulting powder was re-dispersed into water and sonicated for three hours in an ultrasound bath (Autoscience 10200B, with a frequency of 50–60 Hz) in order to obtain GO. RGO was obtained by adding 1 g hexamethylentetramine (HMTA, Sigma Aldrich) to the homogeneous graphene oxide dispersion and kept under stirring at 100 °C for ten hours [5]. The RGO was filtered and washed with distilled water until neutral pH and dried overnight at 60 °C. The GO powder was obtained from the sonicated dispersion after filtering and drying at 60 °C.

### 3.2. Polymer Solution Preparation

The polymer solution was prepared by dissolving 5.5 g of nylon 6,6 (Ultramid^®^ A3K, BASF) in 20 g formic acid (88%, Sigma-Aldrich) for two hours at 70 °C. Afterwards the nanomaterial was added and the solution was stirred for two hours more. Three nanomaterial contents were chosen: 0.1, 0.5 and 1 wt %. The solution concentration was sufficient for the successful electrospinning of nanofibre films.

### 3.3. Electrospinning

Nylon 6,6 electrospun nanocomposites were prepared by electrospinning process. The nylon 6,6/nanomaterial solution was fed into a 5 mL syringe (21 gauge, 1ʺ needle). The flow rate of the solution was controlled using a syringe pump (KDScientific 101) and kept constant at 5 mL/h. A voltage of 20 kV was applied directly to the needle; the tip-collector distance was 15 cm. A copper plate was used.

### 3.4. Carbon Materials Characterisation

FTIR spectroscopy was carried out on a Bruker Optics Vector 33 spectrometer (resolution 1 cm^−1^). Raman spectra were obtained using a Dylor LabRam II equipment with an excitation line of 632.8 nm (resolution 1 cm^−1^). TEM micrographs were taken on a JEOL TEM 1010 microscope operating at 80 kV.

### 3.5. Electrospun Nanocomposites Characterisation

FTIR spectroscopy was carried out in a Perkin Elmer Spectrum 100 spectrometer (resolution 4 cm^−1^). Raman measurements were performed on a Bruker Optics Senterra dispersive spectrometer with excitation laser beam of 785 nm (resolution 4 cm^−1^). The morphology of the nanofibres was characterised by SEM using a JEOL JSM 6060 microscope at 28 kV accelerating voltage.

A Perkin Elmer DSC-7 equipment, was used to determine the crystallinity and the nucleating behaviour of electrospun nanocomposites. This was calibrated with an Indium standard using a constant nitrogen flow both in the sample and in the reference chambers. All samples weighed approximately 6 mg and were sealed within aluminium pans. The samples were heated up to 280 °C for five min and then they were cooled at 10 °C/min.

WAXD diffractograms were obtained in PANalytical X’Pert Pro X-ray diffraction equipment with Cu Kα radiation (*k* = 0.154 nm). The scanning rate was 0.05°/s.

Thermo-mechanical properties of the nanocomposites were measured using a DMA, TA Instruments DMA 2980. The analyses were performed on samples of 30 × 5 × 0.05 mm^3^ under tension film mode in a temperature range of room temperature to 200 °C at a frequency of 1 Hz and a heating rate of 5°/min.

## 4. Conclusions

Carbon based nylon 6,6 electrospun nanofibres were obtained in order to study for the first time the effects of the addition of 1D and 2D nanometric carbon when processed at the same conditions. The experiments were based on the same polymer, processing approach and concentrations of both carbon nanomaterials. The structure of nanomaterials and the functionalisation were found to play an important role in the properties of the nanocomposites. Both 1D CNT and 2D graphene based electrospun nanofibres obtained in this study showed enhanced crystallinity and improved reinforcing effect compared to pure polymer. The features found by FTIR spectroscopy demonstrated that functionalisation increases the interfacial adhesion between polymer and nanofillers and that it also increased the crystalline hydrogen-bonded chains. Crystallisation thermograms displayed the superior ability of CNTs to induce crystallisation in nylon 6,6. On the other hand the heating thermograms of the graphene based nanofibres showed higher crystallinity. The two-dimensional nature of graphene provided a larger surface area that favoured higher crystallinity compared to CNTs in the electrospun fibres. Lower crystal size values were obtained due to the structure of graphene and the different crystallisation process of this material. In addition interface links in the nanocomposites were enhanced by functionalisation of carbon nanomaterials and the functional groups of nylon 6,6. These factors had a significant influence in the increase of the thermo-mechanical properties of the nanofibres.
